# Vegetarianism, low meat consumption and the risk of colorectal cancer in a population based cohort study

**DOI:** 10.1038/srep13484

**Published:** 2015-08-28

**Authors:** Anne M. J. Gilsing, Leo J. Schouten, R. Alexandra Goldbohm, Pieter C. Dagnelie, Piet A. van den Brandt, Matty P. Weijenberg

**Affiliations:** 1 Department of Epidemiology, GROW-School for Oncology and Developmental Biology, Maastricht University, Maastricht, The Netherlands; 2 TNO, Leiden, The Netherlands; 3 Department of Epidemiology, CAPHRI School of Public Health and Primary Care, Maastricht University, Maastricht, The Netherlands

## Abstract

To study how a vegetarian or low meat diet influences the risk of colorectal cancer compared to a high meat diet, and to assess the explanatory role of factors associated with these diets. In the Netherlands Cohort Study – Meat Investigation Cohort (NLCS-MIC) (cohort of 10,210 individuals including 1040 self-defined vegetarians), subjects completed a baseline questionnaire in 1986, based on which they were classified into vegetarians (n = 635), pescetarians (n = 360), 1 day/week- (n = 1259), 2–5 day/week- (n = 2703), and 6-7 day/week meat consumers (n = 5253). After 20.3 years of follow-up, 437 colorectal cancer cases (307 colon, 92 rectal) were available. A non-significantly decreased risk of CRC for vegetarians, pescetarians, and 1 day/week compared to 6-7 day/week meat consumers was observed (age/sex adjusted Hazard Ratios (HR): 0.73(0.47–1.13), 0.80(0.47–1.39), and 0.72(0.52–1.00), respectively). Most of the differences in HR between these groups could be explained by intake of dietary fiber and soy products. Other (non-)dietary factors characteristic for a vegetarian or low meat diet had negligible individual effects, but attenuated the HRs towards the null when combined. Vegetarians, pescetarians, and 1 day/week meat eaters showed a non-significantly decreased risk of colorectal cancer compared to 6-7 day/week meat consumers, mainly due to differences in dietary pattern other than meat intake.

Only a few prospective cohort studies specifically set out to study colorectal cancer incidence in vegetarians. The Oxford Vegetarian study, the EPIC-Oxford study, and the Adventists Health Study (AHS) I and II, intentionally included a large proportion of vegetarians, but yielded inconsistent and mixed results^1^,^2^,^3^,^4^,^5^,^6^.

Some of the inconsistency in findings may be owing to differences in sampling strategies between studies with some studies also recruiting more health-conscious non-vegetarians. Other inconsistencies may arise as a result of differences in definition and operationalization of vegetarianism, and the usefulness and validity of self-defined vegetarianism in etiological studies of cancer remains unclear. Several additional questions concerning the effect of vegetarian diets on colorectal cancer stay unanswered and require further investigation. Although it is speculated that risk factors for colon and rectal cancer may vary^7^,^8^, anatomic subsite-specific effects of vegetarian diets have only been described in the AHS II^6^. Moreover, it is unclear whether the association between vegetarianism and colorectal cancer risk differs by time of adherence to the dietary regimen.

Within the existing studies of meat, vegetarianism and colorectal cancer risk, little to no attention has been paid to the lower end of the meat consumption spectrum. It would be interesting to address whether complete abstinence of meat is associated with a lower colorectal cancer risk than very low meat consumption, or vice versa. Moreover, it is likely that possible beneficial effects of vegetarianism and low meat consumption can, apart from the (near) abstinence of meat, also be attributed to other dietary and lifestyle factors that characterize these diets^9^,^10^. Yet, little is known about the potential explanatory role of these multiple connected factors in the associations of vegetarian and low meat diets with colorectal cancer risk.

We investigated the association between vegetarianism, (low) meat consumption and colorectal cancer risk with special focus on colorectal sub-locations, the time of adherence to the dietary regimen, the validity of self-defined vegetarianism, and the contribution of individual dietary and lifestyle factors within the “Netherlands Cohort Study-Meat Investigation Cohort” (NLCS-MIC). This population based cohort includes a considerable number of vegetarians, pescetarians and low meat consumers resulting in a wide distribution of dietary and lifestyle characteristics that should facilitate the identification of associations with colorectal cancer risk.

## Results

The distribution of demographic and (non-) dietary characteristics according to meat consumption group has been described previously^9^. The percentage of men and supplement users was higher in rectal cancer cases than in non-cases (*P* < 0.05) (Table 1). The percentage of vegetarians was lowest in rectal cancer cases while the proportion of 6-7 day/week meat consumers was highest in this group. The latter is also reflected in a higher total energy intake, and consumption of pork and processed meat among the rectal cancer cases compared to non-cases (*P* < 0.05). However, non-cases had higher daily intakes of milk and cheese, but lower intakes of alcohol than cases (*P *< 0.05). Rectum cancer cases consumed more eggs than non-cases.

Table 2 shows hazard rate ratios and 95% confidence intervals for colorectal, colon and rectal cancer according to meat consumption group, vegetarian status and meat consumption status, adjusted for age and sex alone and with further adjustment for confounders. There was no evidence of an interaction by sex for any of the comparisons made (*P*interaction > 0.05). A borderline statistically significant reduced risk of colorectal cancer for 1 day/week meat consumers was found when compared to 6-7 days/week meat consumers (HR = 0.72, 95% CI:0.52–1.00). Further adjustment for confounding attenuated this association (HR = 0.77, 95% CI:0.55–1.08). A similar though not statistically significant pattern was observed for colon cancer, while the HR for rectal cancer was lowest among the vegetarians (HR = 0.21, 95% CI:0.03–1.55 and increased with increasing frequency of meat intake (*P*trend = 0.03 (age-/energy-adjusted model)). There was only one rectal cancer case that adhered to a vegetarian diet. The inverse association between risk of colorectal cancer and confirmed or self-reported vegetarian status did not reach statistical significance, but was notably attenuated using self-definition (HR = 0.78, 95% CI:0.51–1.20 and HR = 0.91, 95% CI:0.66–1.25, respectively). Similar findings were observed for rectal cancer, whereas HRs around 1 were observed for colon cancer risk. Although the effect of the five meat consumption groups did not differ between colon and rectal cancer cases, the effect of vegetarian status (confirmed versus complementary group of non-confirmed vegetarians) was significantly different between both endpoints (*P*heterogeneity = 0.02). When vegetarians, pescetarians and 1 day/week meat consumers were combined and compared against individuals eating meat >1 day/week, a statistically significant 25% reduction in risk of colorectal cancer was observed, which was no longer significant after adjustment for confounders.

No striking differences between short term and long term adherence (≤10 years versus >10 years) to a vegetarian or low meat diet were observed regarding colorectal cancer risk (data not shown). Within the colon, the risks for proximal or distal tumors were not statistically different for either meat consumption group (*P*heterogeneity = 0.26) or confirmed vegetarian status (*P*heterogeneity = 0.06) (data not shown). Estimates for distal colon cancer were most comparable to those observed for rectal cancer (data not shown). Moreover, in a lag analysis excluding the first 2 years of follow-up (408 colorectal cancer cases), the findings for meat consumption group and vegetarian status did not change appreciably (data not shown).

Table 3 shows the percent change in HR for overall colorectal cancer risk across the meat consumption groups, firstly adjusted for age and sex, then further adjusted for energy and each food group, or lifestyle factor in turn. Fiber and soy product intake contributed most to the observed inverse risk of colorectal cancer when comparing vegetarians, pescetarians, and 1 day-week meat eaters to 6-7 day-week meat. When all confounding dietary and lifestyle factors were added together in one model simultaneously, the HRs approached the null. Comparable patterns were observed when examining colon and rectal cancer separately. In addition, we observed evidence that substituting 5% of energy from protein from meat by 5% of energy from dairy protein was associated with a 24% reduced risk of colorectal cancer, after adjustment for confounding variables (*P *= 0.055) (data not shown). Substituting protein from meat by other sources of protein (e.g. protein from plant sources, eggs or fish) had no statistically significant effect on risk for colorectal cancer (data not shown).

No clear association was observed when the relation was examined between dietary intake of individual meat types and the risk of overall colorectal cancer (Table 4). Similar findings were observed for colon cancer risk. When restricting analyses to the rectal cancer subtype, we observed HRs close to 2 (not statistically significant) across all tertiles of total fresh meat, fresh red meat, and pork consumers when compared to non-consumers. A statistically significant increased risk for rectal cancer was observed with each 25 g-day increment in processed meat intake (HR: 1.36, 95% CI:1.01–1.81) as well as a significant trend across categories of intake (*P*trend = 0.008).

## Discussion

Results from this prospective cohort study showed a modest, non-significantly decreased risk of colorectal cancer for vegetarians, pescetarians, and 1 day/week meat eaters compared to 6-7 day/week meat consumers.

Although the risk of colorectal and especially rectal cancer was lower in vegetarians than in non-vegetarians, this was not statistically significant. Nonetheless, our null findings are in line with a report from the Oxford Vegetarian Study^1^, and a pooled analysis combining data from two prospective studies in the United Kingdom^3^. Colorectal cancer mortality also did not differ between vegetarians and non-vegetarians in a collaborative analysis of five protective cohort studies^11^. In contrast, the colorectal cancer rate was higher among vegetarians than non-vegetarians in the EPIC-Oxford study, but this cohort included a relative health conscious population of non-vegetarians^4^. The Adventist Health Study-I reported that vegetarians had a significantly lower risk of colorectal cancer than non-vegetarians^2^ (average meat consumption of ~3.5 servings/week). A recent publication of the AHS-II confirmed these findings and indicated that especially pescetarians were at a reduced risk of colorectal cancer^6^. The low rates of tobacco use and limited consumption of alcohol among this population reduces the likelihood of confounding by these factors.

We examined to what extent the associations between the diet-groups could be explained by other factors than the frequency of meat consumption. After adjusting the analyses for major risk factors of colorectal cancer such as total cigarette smoking, alcohol consumption, BMI, and physical activity, our results attenuated slightly suggesting that the lower risk observed in vegetarians and low meat consumers was only partly due to these lifestyle differences between meat consumption groups. While it has been proposed that the lower disease risk in vegetarians may be explained by selective factors related to who chooses to become and remain a vegetarian^12^, adjustment for e.g. level of education did not change the estimates notably either. In the AHS II, effect estimates for colorectal and colon cancer did change notably after correction for lifestyle factors, while estimates for rectal cancer became stronger. The AHS-II did not examine the effect of dietary factor other than fiber on their risk estimates. In our analyses, fiber and soy product intake accounted for the greatest change in HRs when comparing vegetarians and low meat consumers to 6-7 day/week meat consumers. Although soy intake levels in our study are low and the evidence for a protective effect are inconsistent^13^, the WCRF expert panel concluded that there is convincing evidence that dietary fiber protects against colorectal cancer^14^. All other dietary factors that characterize a vegetarian and low meat diet had negligible individual effects, but when combined they attenuated the risk estimates, by at least 17 percent, approaching the null. This suggests that the effect of a single food group or lifestyle variable may be too small to detect, but the cumulative cancer preventive effects of multiple connected dietary and non-dietary factors may be sufficiently large to be demonstrable^15^.

No study previously examined the effect of very low meat diets on colorectal cancer incidence. Our findings suggest that especially very low meat consumers may have a reduced risk of colorectal cancer compared to frequent meat consumers which was mainly observed for the colon cancer subgroup. Analyses from the Adventists Health Study-II suggest that, after an average of 4 years follow-up, compared to non-vegetarians the lowest risk of all gastro-intestinal (GI) cancers combined was observed among semi-vegetarians who ate red meat, poultry or fish once/month to once/week (age-adjusted HR = 0.64; 95% CI:0.42–0.99)^5^. However, it is likely that this observation was driven by other GI cancers since the more recent findings did not find indications that semi-vegetarians were at a reduced risk for colorectal cancer in the AHS II^6^. Whether a vegetarian diet is nutritionally adequate remains equivocal^16^, and is outside the scope of this paper. It is however, interesting to observe that a strict vegetarian diet does not seem to have an additional colorectal cancer preventive effect over 1 day/week meat consumption in our population. Nonetheless, future studies with larger numbers of no and low meat consumers should replicate these findings.

Individuals adhering to a no or low meat diet often replace the meat in their diet with other (protein rich) food groups. Our observation that these individuals may have a lower colorectal cancer risk than high meat consumers is supported by our findings that substituting protein from meat with an equal percentage of energy from dairy protein significantly reduced the risk of colorectal cancer. In fact, dairy products have been hypothesized to protect against colorectal cancer risk due to their high calcium content^14^,^17^.

No universally accepted definition for vegetarianism exists and the operationalization of vegetarianism differs between studies, and the usefulness and validity of self-reported vegetarianism in etiological studies remains unclear. For this purpose, we examined the association between vegetarianism and colorectal cancer risk using both self-definition and FFQ confirmed vegetarian status. Although both methods of classification yielded statistically non-significant protective effects, the association was considerable stronger for confirmed vegetarians than for non-vegetarians, suggesting that some attenuation occurs when merely relying on self-definition for classification purposes.

Based on our sub-site analyses, all the risk estimates appeared to be more strongly associated with rectal tumors, except for low meat consumption, which mainly decreased the risk of colon, but not rectal, cancer. Although the risk estimates for vegetarian patterns in the AHS-II were comparable for the colon and rectum subsite, they only reached statistical significance in the colon, potentially due to smaller number of rectal cancer cases^6^. Previous studies suggest that meat, subtypes of meat, and meat related carcinogens may act differently at various locations in the colorectum^8^,^18^, possibly as a result of e.g. sub-site differences in bacterial composition and bacterial metabolic capacity, enzyme activity, and transit time^7^,^19^,^20^,^21^. Although the number of especially rectal cancer cases among the vegetarians and low meat consumers in our population was low, our observation that distal colonic tumors exerted a similar pattern of association, strengthens our findings.

As a result of our sampling strategy, our population has a large contrast in meat intake which should aid the further specification of associations between subtypes of meat and colorectal cancer risk. We found no clear association between total fresh and fresh red meat intake and colorectal cancer risk. Processed meat was only associated with rectal cancer; again, comparable findings were observed for distal colon, but not for proximal colon cancer risk. Although processed meat is widely recognized risk factor for colorectal cancer^14^,^22^, this endpoint heterogeneity was not observed in a recent meta-analysis^22^. Processed meat is known to be the major source of human exposure to nitrite, and contains all the necessary precursors for *N-* nitroso compound (NOC) formation^23^; both have been specifically associated with increase rectal cancer risk only^18^,^24^,^25^.

NLCS-MIC was specifically designed for analyzing the effect of no and low meat dietary habits on cancer risk. We performed a multi-perspective approach to study meat consumption and cancer risk in our analyses looking both at meat consumption groups and individual meat items and some findings may be due to chance. Future studies are needed to confirm our findings. The NLCS attempted to enlarge the exposure contrast in the cohort by extra recruitment of vegetarian subjects^26^ vegetarian dietary patterns were taken into consideration when designing the FFQ, and vegetarian status was taken into account for nutrient calculation of composite recipes. Nonetheless, our analyses have been performed using baseline FFQ data resulting in an inability to assess and account for changes in dietary intakes over time. However, the validity of the FFQ has been tested and shown to be representative for dietary habits over a period of at least 5 years^27^,^28^. Although we have information on time that people had adhered to their special dietary regimen at the start of follow-up (1986), stratified analyses (≤10 years versus >10 years) yielded similar findings, possibly due to small numbers. The prospective design eliminates the potential for recall bias, and the nearly complete follow-up makes selection bias unlikely. Detailed information on diet and potential risk factors of colorectal cancer enabled us to control for most known risk factors, although misclassification of exposure may have occurred.

In summary, vegetarians, pescetarians, and especially 1 day/week meat eaters showed a modest, non-significantly decreased risk of colorectal cancer compared to 6-7 day/week meat consumers, mainly due to differences in dietary patterns other than meat intake.

## Methods

### Study population and cancer follow-up

The NLCS ‘Meat Investigation Cohort’ (NLCS-MIC) is an analytical cohort embedded within the ongoing prospective Netherlands Cohort Study (NLCS). The total NLCS study was initiated in September 1986 and includes 120,852 men and women aged 55–69 years at baseline, largely originating from 204 municipalities with computerized population registries. In addition, to increase contrast within the cohort, vegetarians were overrepresented by recruitment through health food shops and magazines. At the start of the study, participants completed a self-administered questionnaire on dietary habits, lifestyle characteristics, medical history, and other potential risk factors for cancer^26^. NLCS-MIC is specifically established within the NLCS to study the health effects of vegetarian and low meat diets. Because the total NLCS-cohort traditionally uses the case-cohort approach for analyses, data was only entered for a random subcohort of 10,000 subjects and all enumerating cancer cases. As a result, NLCS-MIC had to be established by combining the random subcohort with all (self-reported) vegetarians and all individuals that consumed meat only 1 day/week from the total NLCS cohort for whom the data was also entered. All vegetarians and those consuming meat 1 day/week were initially identified based on two items relating to specific dietary regimens that are stated on the first page of the questionnaire that was processed for all 120,852 cohort members: “Do you have any special eating habits?”, and “how many days on average per week do you eat meat?”. The 150 item semi-quantitative food frequency questionnaire (FFQ) was used to accurately categorize NLCS-MIC (n = 11,082) with complete and consistent FFQs into five meat consumption categories: confirmed vegetarians (n = 691) and pescetarians (n = 389), 1 day/week- (n = 1,388), 2–5 days/week- (n=2,965), and 6-7 days/week meat consumers (n = 5,649). We defined vegetarians as individuals who consume a diet void of meat (including vegans, lactoovo-, lacto-, and ovo-vegetarians). Pescetarians do not eat meat but do eat fish. As a consequence of the procedure followed, NLCS-MIC also includes 1,133 self-reported vegetarians of whom 109 reported to consume meat but were not part of the randomly selected subcohort. As a result, these latter individuals are only included in analyses when comparing all self-reported vegetarians (either confirmed or not) to the complementary group of non-(self-reported) vegetarians, and not for all other contrasts. Full details of the study design have been described elsewhere^9^.

The full-cohort approach is used for analyses of NLCS-MIC. NLCS-MIC is being monitored for cancer occurrence by repeated record linkage to the Netherlands Cancer Registry, the Dutch Pathology Registry, and the cause of death registry (Statistics Netherlands), together providing a near 100% coverage^29^. Follow-up for vital status was established by record linkage to the automated municipal population registries and the Central Bureau for Genealogy. Less than 1% of the cohort members were lost to follow-up. After 20.3 years of follow-up and exclusion of prevalent cancer cases at baseline (other than skin cancer), 477 colorectal cancer cases (336 colon (ICD-O codes:153.0–153.7) (184 proximal colon; 142 distal colon and 10 unspecified) and 99 rectum (ICD-O code:154.1)) remained eligible for analyses. Rectosigmoid cancer cases (ICD-O code:154.0) were not evaluated separately because of the small number of cases (n = 42) and the higher risk of misclassification^30^. The NLCS has been approved by the institutional review boards of the TNO Quality of Life Research Institute (Zeist, the Netherlands) and Maastricht University (Maastricht, the Netherlands). All methods were carried out in accordance with the approved guidelines. All cohort members consented to participate in the study by completing and returning the self-administered questionnaire.

## Questionnaire

All participants completed a 150 item semi-quantitative FFQ at baseline, estimating the average frequency and amount of foods and beverages consumed over the previous 12 months. Next to the questions relating to special eating habits and weekly meat consumption frequency that were used for the identification of vegetarians and 1 day/week meat consumers, the questionnaire also assessed the time since the start of any special eating habits and weekly frequency of meat consumption (for 0-1 day/week meat consumers), in years until baseline (1986). In addition, the FFQ contained 14 items on the consumption of meat with the hot meal (mainly fresh meat, including chicken), 5 items on the consumption of meat products used as sandwich fillings, and 3 items on fish consumption (with the hot meal, for lunch, as a snack in between meals). Coding of fresh meat items was based on raw weight to take into account the amount of fat originally present in the meat but eventually ending up in the gravy, which is usually consumed as well. Processed meat was defined as meat items that had undergone some form of preservation (mostly cured (i.e. treated with nitrite/nitrate salt, sometimes smoked and/or fermented)).

A validation study conducted in a subgroup of the cohort two years after the baseline measurement indicated that the Spearman correlation coefficients for meat, meat products and fish as assessed by the questionnaire and those estimated from the 9-day record were 0.46, 0.54 and 0.53 respectively. The number of vegetarians and 1 day/week meat consumers was too low in this validation sample to assess a correlation in these extremes^28^.

## Statistical analyses

We estimated the association between meat consumption group (confirmed vegetarian, pescetarian, 1 day/week-, 2–5 days/week-, and 6-7 days/week meat consumers (reference group)) and the risk of colorectal cancer. In addition, the association with self-reported vegetarian status (self-reported versus complementary group of non-self-reported-vegetarians) and confirmed vegetarian status (confirmed vegetarian versus complementary group of non-vegetarians) was examined. To increase power, vegetarians and fish eaters were combined in an overlapping category of non-meat consumers to examine whether their risk of colorectal cancer differed from the complementary group of individuals who do consume meat. To assess to what extent these associations can (partially) be explained by other dietary and lifestyle variables (e.g. smoking, physical activity, BMI and level of education), we calculated the difference in risk estimate, firstly adjusting for age and sex, then further adjusting for energy and each food group, or lifestyle factor in turn. Moreover, the association with meat consumption group and confirmed vegetarians status was stratified by duration of adherence to the specific diet (≤10 years, >10 years).

Individuals adhering to a no or low meat diet often replace the meat in their diet with other protein-rich food groups. Using nutrient density substitution models and meat protein as a proxy for meat intake, we examined the effect of replacing one protein subtype for another by including nutrient density variables for all but one protein subtype in a multivariable model along with total protein intake and total energy intake^31^.

The following food groups and foods were also selected for analyses (in g/day): fresh meat (beef, pork, minced meat, chicken, liver), processed meat, fish, fresh red meat (fresh meat without chicken) and beef, pork, minced meat, chicken, and liver as separate types. For the individual meat types, subjects were classified into non-consumers, and tertiles of consumers (non-consumers as reference group), and as continuous variables. The latter were reported in 50 g/day increase for all fresh meat types except liver, and 25 g/day for processed meat and liver intake. For some variables, categories were used instead of quintiles. For liver intake, there was a non-user and a user group (>0 g/day). For both chicken and fish a non-user and 3 user categories (0−<6.6, ≥6.6−<22.5 and ≥22.8 g/day for chicken; 0−<10, ≥10−<20 and ≥20 g/day for fish) were defined.

For all the above described contrasts, age and sex adjusted and multi-variable adjusted hazard rate ratios (HRs) and their corresponding 95% confidence intervals (95% CI) were estimated using Cox proportional hazards models. The proportional hazards assumption was tested using the scaled Schoenfeld residuals. To evaluate whether early symptoms of colorectal cancer before diagnosis could have influenced the results, early cases (diagnosed within 2 years after baseline) were excluded in additional analyses. The covariates included in the multivariate analyses were either a priori selected risk factors of colorectal cancer, or variables that changed the risk estimates for meat consumption group, vegetarian status or total fresh meat intake by 10% or more. The latter criterion was not met for any other than the predefined covariates resulting in a final model including age(years), sex, total energy intake(kcal/day), cigarette smoking (never, ever, current), alcohol consumption (g/day), BMI (kg/m2), non-occupational physical activity (≤30, >30– ≤60, >60−≤90, >90 minutes/day), and level of education (lower vocational, secondary-/medium-vocational, university and higher vocational). The independent contribution of the individual meat categories was examined by constructing addition models that summed to total meat.

To enable comparison, the age and sex-adjusted analyses were restricted to subjects included in multivariable-adjusted, leaving 10,210 cohort members, including 437 colorectal cancer cases (307 colon (129 proximal colon;169 distal colon and 9 unspecified) and 92 rectal) for analyses. Moreover, when analyzing the contrast between self-reported vegetarians and non-vegetarians, an additional 90 self-reported vegetarians that reported to consume meat but were not part of the randomly selected subcohort were also included in analyses (including 7 colorectal cancer cases). Linear trends were evaluated with the Wald test by entering the categorical exposure variables as a continuous term in the Cox regression model.

To test for heterogeneity between the colon and the rectum, and the anatomic subsites of colon cancer (proximal/distal), the competing risks procedure in Stata was used. However, the standard error for the difference of the log-HRs from this procedure assumes independence of both estimated HRs which would overestimate the standard error and thus overestimate the *P* values for their difference. Therefore, these *P* values and the associated confidence intervals were estimated based on a bootstrapping method. Each bootstrap analysis was based on 1,000 replications.

All tests were two-tailed and differences were regarded as statistically significant at *P* < 0.05. All analyses were performed using STATA Statistical Software (Intercooled STATA, version 12; Stata-Corp LP, College Station, TX).

## Additional Information

**How to cite this article**: Gilsing, A. M. J. *et al.* Vegetarianism, low meat consumption and the risk of colorectal cancer in a population based cohort study. *Sci. Rep.* **5**, 13484; doi: 10.1038/srep13484 (2015).

## Figures and Tables

**Table 1:** Baseline characteristics (means or percent) and dietary intakes of exposures of interest of colorectal cancer cases and non-cases in NLCS-MIC, 1986-2006.

Characteristics	Non cases	Colorectal cancer cases	Colon cancer cases^a^	Rectal cancer cases^a^
*N*	9773	437	307	92
Meat consumption group (%)				
** **Vegetarian	6%	5%	6%	1%
** **Pescetarian	4%	3%	4%	2%
** **1 day/wk meat	12%	9%	10%	11%
** **2–5 days/wk meat	26%	27%	27%	25%
** **6-7 days/wk meat	51%	56%	53%	61%
** **Sex (% men)	47%	53%*	50%	64%*
** **Age	61.3 ± 4.2^b^	61.4 ± 4.0	61.5 ± 4.1	61.2 ± 3.8
** **Current smokers (%)	26%	23%	21%	33%
** **BMI mean	24.7 ± 3.2	25.0 ± 3.2	24.9 ± 3.2	25.1 ± 3.1
Physical activity (non-occupational) (%)				
** **<30 min/day	21%	19%	19%	15%
** **30–60 min/day	30%	33%	35%	30%
** **60–90 min/day	23%	19%	21%	16%
** **>90 min/day	26%	28%	25%	38%
Level of education (%)				
** **Low	46%	47%	45%	51%
** **Medium	37%	37%	38%	37%
** **High	17%	16%	17%	12%
** **Supplement use (% users)	34%	31%	36%	22%*
** **Energy (kcal)	1888 ± 519	1919 ± 512	1888 ± 515	2032 ± 503*
** **Fiber (g)	28.2 ± 7.6	27.8 ± 7.2	27.8 ± 7.3	27.9 ± 6.5
** **Alcohol (g)	9.3 ± 13.8	11.8 ± 16.4*	11.9 ± 16.7*	12.3 ± 17.2*
** **Total fresh meat (g)^c^	81.1 ± 52.8	83.6 ± 48.1	80.9 ± 48.6	88.2 ± 48.4
** ** Fresh red meat (g)^d^	70.6 ± 49.2	73.1 ± 45.5	70.7 ± 46.0	76.5 ± 44.3
** **Beef (g)	21.6 ± 23.9	22.1 ± 21.3	22.4 ± 21.9	19.5 ± 19.0
** **Pork (g)	30.5 ± 29.8	32.4 ± 29.0	29.9 ± 27.6	36.7 ± 28.6*
** **Minced meat (g)	14.9 ± 16.3	15.3 ± 15.0	15.1 ± 14.8	16.7 ± 16.7
** **Liver (g)	1.6 ± 4.0	1.6 ± 3.7	1.5 ± 3.6	1.9 ± 4.1
** **Chicken (g)	11.3 ± 14.8	11.2 ± 14.1	10.9 ± 12.3	12.3 ± 15.1
** **Processed meat (g)	11.0 ± 14.3	12.3 ± 14.4	11.4 ± 13.6	16.3 ± 17.8*
** **Fish (g)	13.1 ± 18.1	12.6 ± 14.9	13.3 ± 15.8	13.3 ± 12.9
** **Vegetables (g)	199 ± 89	200 ± 85	200 ± 88	204 ± 81
** **Fruits (g)	184 ± 128	175 ± 115	176 ± 115	168 ± 110
** **Pulses (g)	10.0 ± 16.7	10.1 ± 17.1	9.9 ± 13.3	9.5 ± 13.9
** **Soy products (g)	3.1 ± 17.8	2.0 ± 9.4	2.7 ± 9.9	0.4 ± 1.5
** **Milk (g)	312 ± 208	285 ± 189*	288 ± 193*	284 ± 188
** **Cheese (g)	25.9 ± 22.6	24.2 ± 20.8	23.1 ± 20.0*	30.4 ± 24.4
** **Eggs (g)	15.6 ± 11.7	16.2 ± 11.1	15.3 ± 10.0	18.7 ± 14.7*

^a^Colon and rectal cases do not sum to total colorectal cancer cases because rectosigmoid cases were not evaluated separately.

^b^mean ± SD, all such values.

^c^Intake based on raw meat weight.

^d^Includes beef, pork, minced meat, liver and other meat.

^*^Statistically significant different from non-cases (using the χ2 test for categorical variables and ANOVA for continuous variables).

**Table 2:** Hazard rate ratios (HR) and 95% confidence intervals (CI) for colorectal cancer according to meat consumption group, vegetarian status and meat consumption status.

Factor	Category	Person years	Colorectal cancer					Colon cancer					Rectal cancer					
			Cases	HR	(95%CI)^a^	HR	(95%CI)^b^	Cases	HR	(95%CI)^a^	HR	(95%CI)	Cases	HR	(95%CI)^a^	HR	(95%CI)^b^	
Meat consumption group																		
	vegetarian	11277	22	0.73	(0.47–1.13)	0.83	(0.53–1.31)	19	0.91	(0.57–1.47)	1.01	(0.62–1.66)	1	0.16	(0.02–1.15)	0.21	(0.03–1.55)	
	pescetarian	6429	14	0.80	(0.47–1.39)	0.88	(0.51—1.51)	11	0.92	(0.50–1.70)	0.96	(0.52–1.80)	2	0.52	(0.13–2.14)	0.68	(0.16–2.84)	
	1 day/wk meat	21451	41	0.72	(0.52-1.00)	0.77	(0.55–1.08)	30	0.76	(0.51–1.12)	0.80	(0.54–1.20)	10	0.83	(0.42–1.62)	0.97	(0.48–1.95)	
	2-5 day/wk meat	43360	116	0.93	(0.75–1.16)	0.95	(0.76–1.19)	82	0.96	(0.74–1.26)	0.99	(0.76–1.29)	23	0.84	(0.51–1.36)	0.87	(0.53–1.42)	
	6-7 day/wk meat	87356	244	1 (ref)		1 (ref)		165	1 (ref)		1 (ref)		56	1 (ref)		1 (ref)		
	*P*_trend_			0.04		0.19			0.36		0.7			0.03		0.14		
Vegetarianism	vegetarian^c^	11277	22	0.78	(0.51–1.20)	0.89	(0.58–1.39)	19	0.95	(0.60–1.52)	1.06	(0.65–1.70)	1	0.17	(0.02–1.25)	0.22	(0.03–1.63)	
	non vegetarian	160598	415	1 (ref)		1 (ref)		288	1 (ref)		1 (ref)		91	1 (ref)		1 (ref)		
	self-defined vegetarian^d^	18272	42	0.91	(0.66–1.25)	1.04	(0.74–1.44)	31	0.96	(0.66–1.39)	1.06	(0.72–1.55)	5	0.50	(0.20–1.24)	0.65	(0.26–1.63)	
	non self-defined vegetarian	154961	402	1 (ref)		1 (ref)		278	1 (ref)		1 (ref)		89	1 (ref)		1 (ref)		
Meat consumption	No meat consumers	17706	36	0.81	(0.57–1.14)	0.90	(0.63–1.29)	30	0.96	(0.66–1.40)	1.04	(0.70–1.53)	3	0.32	(0.10–1.00)	0.41	(0.13–1.32)	
	Meat consumers	154169	401	1 (ref)		1 (ref)		277	1 (ref)		1 (ref)		89	1 (ref)		1 (ref)		
	≤1 day/wk meat	39158	77	0.75	(0.58–0.97)	0.82	(0.63–1.06)	60	0.84	(0.62–1.12)	0.89	(0.66–1.20)	13	0.62	(0.34–1.12)	0.77	(0.41–1.42)	
	>1 day/wk meat	132717	360	1 (ref)		1 (ref)		247	1 (ref)		1 (ref)		79	1 (ref)		1 (ref)		

^a^Adjusted for age (yrs) and sex.

^b^Adjusted for age (yrs), sex, total energy intake (kcal), cigarette smoking (never, ever, current), alcohol consumption (g/day), BMI (kg/m2), non-occupational physical activity (<30, 30–60, 60–90, >90 min/d), and level of education (lower vocational, second and medium vocational, university and higher vocational).

^c^Confirmed vegetarians based on the extensive Food Frequency Questionnaire (defined as individuals who consume a diet void of meat).

^d^NLCS-MIC includes 1,133 self-defined vegetarians of whom 109 reported to consume meat but were not part of the randomly selected subcohort. These individuals are only included in analyses when comparing all self-defined vegetarians (either confirmed or not) to non-self-defined vegetarians, and not for all other contrasts.

**Table 3:** Difference in Hazard rate ratios (HR) for colorectal cancer between vegetarians, pescetarians, 1 day/wk meat and and 6-7 days/wk meat consumers after adjustment for individual dietary and lifestyle factors.

	Colorectal cancer						
	vegetarian		pescetarian		1 day/wk meat		6–7 day/wk meat
Factor adjusted for^a^	HR	% change in HR	HR	% change in HR	HR	% change in HR	HR
Age and sex	0.73	−	0.80	−	0.72	−	1 (ref)
Dietary Factors							
Energy (kcal)	0.73	0%	0.80	0%	0.71	−1%	1 (ref)
Energy + alcohol (g)	0.77	4%	0.82	2%	0.74	2%	1 (ref)
Energy + fiber (g)	0.82	9%	0.89	9%	0.76	4%	1 (ref)
Energy + fruits (g)	0.75	2%	0.82	2%	0.72	0%	1 (ref)
Energy + vegetables (g)	0.72	−1%	0.79	−1%	0.71	−1%	1 (ref)
Energy + pulses (g)	0.71	−2%	0.78	−2%	0.70	−2%	1 (ref)
Energy + soy products (g)	0.78	5%	0.87	7%	0.73	1%	1 (ref)
Energy + milk (g)	0.76	3%	0.83	3%	0.74	2%	1 (ref)
Energy + cheese (g)	0.77	4%	0.84	4%	0.74	2%	1 (ref)
Energy + eggs (g)	0.73	0%	0.80	0%	0.71	−1%	1 (ref)
Energy + Supplement use (none, 1 sup, ≥2 sup)	0.75	2%	0.83	3%	0.73	1%	1 (ref)
Full model including dietary factors^b^	0.99	26%	1.04	24%	0.86	14%	1 (ref)
Lifestyle Factors							
Smoking^c^	0.72	−1%	0.79	−1%	0.71	−1%	1 (ref)
Non occupational physical activity^d^	0.73	0%	0.81	1%	0.72	0%	1 (ref)
BMI (kg/m2)	0.77	4%	0.84	4%	0.74	2%	1 (ref)
Level of education^e^	0.75	2%	0.83	3%	0.73	1%	1 (ref)
Full model including dietary and lifestyle factors^b^	1.06	33%	1.11	31%	0.89	17%	1 (ref)

^a^All adjusted for age and sex.

^b^Including all the above listed variables.

^c^Smoking categories: never, ever, and current smokers.

^d^Non occupational physical activity categories: <30, 30–60, 60–90, >90 min/d.

^e^Level of education categories: lower vocational, second and medium vocational, university and higher vocational.

**Table 4:** Hazard rate ratios (HR) and 95% confidence intervals (CI) for colorectal cancer according to sex-specific quintiles and categories of intake of fresh meat, types of fresh meat, and processed meat.

Food item	Median intake		Person years	Colorectal cancer					Colon cancer					Rectal cancer				
	Men	Women		Cases	HR	(95%CI)^a^	HR	(95%CI)^b^	Cases	HR	(95%CI)^a^	HR	(95%CI)^b^	Cases	HR	(95%CI)^a^	HR	(95%CI)^b^
Total fresh meat (g/day)^c^																		
Non consumers	0	0	20022	42	1 (ref)		1 (ref)		35	1 (ref)		1 (ref)		4	1 (ref)		1 (ref)	
T1	56.6	30.0	49317	111	1.02	(0.72–1.47)	0.94	(0.66–1.36)	77	0.87	(0.58–1.30)	0.83	(0.55–1.24)	25	2.31	(0.80–6.66)	1.83	(0.63–5.35)
T2	99.5	85.7	51037	161	1.45	(1.03–2.04)	1.26	(0.88–1.80)	115	1.27	(0.87–1.85)	1.14	(0.76–1.71)	36	3.24	(1.15–9.12)	2.25	(0.78–6.50)
T3	142.6	123.8	51499	123	1.11	(0.78–1.57)	0.92	(0.63–1.34)	80	0.88	(0.59–1.31)	0.76	(0.49–1.17)	27	2.42	(0.84–6.92)	1.53	(0.51–4.58)
*P*_trend_					0.28		0.88			0.97		0.46			0.18		0.93	
Continuous (50 g/day intake increment)					1.04	(0.94–1.13)	0.97	(0.88–1.08)		0.99	(0.90–1.11)	0.95	(0.84–1.07)		1.04	(0.89–1.32)	0.94	(0.75–1.17)
Fresh red meat (g/day)^c^																		
Non consumers	0	0	21571	42	1 (ref)		1 (ref)		34	1 (ref)		1 (ref)		5	1 (ref)		1 (ref)	
T1	47.3	24.9	48721	121	1.22	(0.86–1.74)	1.19	(0.83–1.71)	86	1.10	(0.74–1.63)	1.10	(0.73–1.66)	27	2.18	(0.84–5.67)	1.86	(0.70–4.94)
T2	85.5	71.2	50679	138	1.35	(0.96–1.91)	1.26	(0.88–1.82)	94	1.16	(0.78–1.72)	1.12	(0.74–1.70)	32	2.50	(0.97–6.42)	1.94	(0.73–5.16)
T3	127.6	106.9	20904	136	1.33	(0.94–1.88)	1.20	(0.83–1.74)	93	1.15	(0.77–1.70)	1.08	(0.70–1.64)	28	2.18	(0.84–5.65)	1.56	(0.58–4.23)
*P*_trend_					0.12		0.48			0.50		0.85			0.24		0.85	
Continuous (50g/day intake increment)					1.04	(0.95–1.15)	1.00	(0.90–1.11)		1.00	(0.89–1.13)	0.97	(0.86–1.10)		1.04	(0.87–1.32)	0.95	(0.75–1.19)
Beef (g/day)^c^																		
Non consumers	0	0	37701	87	1 (ref)		1 (ref)		66	1 (ref)		1 (ref)		15	1 (ref)		1 (ref)	
T1	8.6	6.3	44979	106	1.00	(0.75–1.32)	0.96	(0.71–1.30)	64	0.80	(0.57–1.14)	0.82	(0.57–1.18)	33	1.72	(0.93–3.16)	1.41	(0.75–2.69)
T2	24.1	19.3	45099	125	1.17	(0.89–1.54)	1.11	(0.82–1.49)	88	1.10	(0.80–1.52)	1.09	(0.77–1.56)	26	1.36	(0.72–2.56)	1.08	(0.54–2.12)
T3	48.9	43.2	44096	119	1.12	(0.85–1.48)	1.04	(0.77–1.40)	89	1.12	(0.81–1.54)	1.09	(0.77–1.54)	18	0.94	(0.48–1.88)	0.72	(0.35–1.48)
*P*_trend_					0.24		0.58			0.17		0.25			0.52		0.13	
Continuous (50 g/day intake increment)					1.02	(0.84–1.25)	0.96	(0.78–1.18)		1.07	(0.85–1.34)	1.02	(0.81–1.30)		0.87	(0.47–1.22)	0.63	(0.38–1.05)
Pork (g/day)^c^																		
Non consumers	0	0	30814	62	1 (ref)		1 (ref)		50	1 (ref)		1 (ref)		8	1 (ref)		1 (ref)	
T1	11.1	6.1	46148	109	1.12	(0.82–1.54)	1.15	(0.82–1.61)	81	1.06	(0.74–1.51)	1.05	(0.72–1.54)	21	1.59	(0.70–3.59)	1.68	(0.71–3.96)
T2	35.0	28.5	47628	148	1.51	(1.12–2.03)	1.50	(1.07–2.11)	103	1.32	(0.94–1.86)	1.29	(0.87–1.90)	31	2.31	(1.06–5.04)	2.28	(0.96–5.41)
T3	66.9	58.3	47285	118	1.21	(0.89–1.65)	1.14	(0.80–1.62)	73	0.95	(0.66–1.36)	0.88	(0.58–1.33)	32	2.40	(1.10–5.21)	2.08	(0.88–4.91)
*P*_trend_					0.10		0.45			0.98		0.55			0.01		0.12	
Continuous (50 g/day intake increment)					1.10	(0.94–1.28)	1.02	(0.86–1.21)		0.97	(0.80–1.18)	0.91	(0.73–1.13)		1.14	(0.95–1.76)	1.07	(0.76–1.50)
Minced meat (g/day)^c^																		
Non consumers	0	0	40870	81	1 (ref)		1 (ref)		62	1 (ref)		1 (ref)		14	1 (ref)		1 (ref)	
T1	6.8	5.5	43368	112	1.27	(0.95–1.69)	1.27	(0.94–1.71)	78	1.17	(0.84–1.63)	1.21	(0.85–1.73)	24	1.51	(0.78–2.92)	1.42	(0.71–2.82)
T2	16.7	14.0	44180	137	1.53	(1.16–2.02)	1.50	(1.11–2.04)	91	1.35	(0.98–1.87)	1.42	(0.99–2.03)	29	1.78	(0.94–3.37)	1.62	(0.81–3.25)
T3	34.0	29.0	43456	107	1.21	(0.91–1.63)	1.18	(0.86–1.62)	76	1.15	(0.82–1.61)	1.20	(0.82–1.74)	25	1.57	(0.81–3.02)	1.37	(0.68–2.80)
*P*_trend_					0.10		0.32			0.31		0.33			0.18		0.45	
Continuous (50 g/day intake increment)					1.06	(0.80–1.40)	0.97	(0.72–1.32)		1.05	(0.75–1.48)	1.03	(0.72–1.48)		1.11	(0.69–2.17)	1.06	(0.57–1.97)
Liver(g/day)^c^																		
Non consumers	0	0	120706	300	1 (ref)		1 (ref)		214	1 (ref)		1 (ref)		63	1 (ref)		1 (ref)	
Consumers	4.1	3.3	51169	137	1.08	(0.88–1.33)	1.08	(0.87–1.33)	93	1.04	(0.82–1.33)	1.05	(0.82–1.37)	29	1.05	(0.68–1.64)	1.01	(0.64–1.59)
Continuous (25 g/day intake increment)					1.08	(0.60–1.94)	1.10	(0.60–2.01)		0.87	(0.41–1.84)	0.92	(0.42–2.00)		1.50	(0.50–4.54)	1.34	(0.42–4.27)
Chicken^c^,^d^																		
Non consumers	0	0	56451	137	1 (ref)		1 (ref)		101	1 (ref)		1 (ref)		24	1 (ref)		1 (ref)	
C1	5.3	5.3	39581	97	1.00	(0.77–1.30)	0.98	(0.75–1.28)	68	0.96	(0.70–1.30)	0.96	(0.70–1.31)	23	1.34	(0.76–2.38)	1.30	(0.73–2.33)
C2	13.2	13.2	36198	95	1.07	(0.83–1.39)	1.01	(0.77–1.33)	66	1.02	(0.75–1.39)	0.98	(0.71–1.35)	17	1.07	(0.57–1.99)	1.00	(0.53–1.91)
C3	22.8	22.8	39645	108	1.10	(0.86–1.42)	1.04	(0.80–1.35)	72	1.01	(0.74–1.36)	0.96	(0.71–1.32)	28	1.58	(0.91–2.73)	1.47	(0.84–2.58)
*P*_trend_					0.40		0.73			0.89		0.85			0.17		0.27	
Continuous (50 g/day intake increment)					0.96	(0.69–1.33)	0.89	(0.64–1.25)		0.92	(0.62–1.35)	0.86	(0.57–1.28)		1.09	(0.63–2.25)	1.08	(0.56–2.10)
Processed meat (g/day)																		
Non consumers	0	0	43461	98	1 (ref)		1 (ref)		74	1 (ref)		1 (ref)		16	1 (ref)		1 (ref)	
T1	3.9	2.3	42977	100	0.99	(0.75–1.30)	0.97	(0.72–1.30)	77	1.02	(0.74–1.41)	1.02	(0.73–1.44)	12	0.68	(0.32–1.45)	0.69	(0.32–1.52)
T2	13.2	8.4	43249	117	1.16	(0.89–1.52)	1.13	(0.84–1.52)	74	0.99	(0.72–1.37)	1.00	(0.70–1.43)	31	1.77	(0.96–3.24)	1.72	(0.88–3.36)
T3	30.8	20.3	42188	122	1.25	(0.95–1.64)	1.24	(0.91–1.69)	82	1.14	(0.83–1.56)	1.17	(0.81–1.69)	33	1.95	(1.07–3.55)	1.88	(0.94–3.75)
*P*_trend_					0.05		0.09			0.48		0.41			0.002		0.008	
Continuous (25 g/day intake increment)					1.13	(0.97–1.31)	1.12	(0.95–1.33)		1.05	(0.87–1.28)	1.08	(0.87–1.34)		1.45	(1.12–1.87)	1.36	(1.01–1.81)
Fish^e^																		
Non consumers	0	0	53879	113	1 (ref)		1 (ref)			1 (ref)		1 (ref)		18	1 (ref)		1 (ref)	
C1	4.6	4.6	37645	112	1.41	(1.09–1.83)	1.38	(1.06–1.79)	86	1.34	(0.99–1.82)	1.30	(0.96–1.77)	24	1.85	(1.00–3.41)	1.78	(0.96–3.29)
C2	14.8	14.8	48056	128	1.25	(0.97–1.61)	1.19	(0.92–1.54)	80	1.09	(0.80–1.47)	1.03	(0.76–1.39)	31	1.85	(1.03–3.30)	1.81	(1.01–3.25)
C3	32.8	30.2	32295	84	1.21	(0.91–1.61)	1.16	(0.87–1.55)	84	1.10	(0.78–1.54)	1.06	(0.75–1.49)	19	1.63	(0.86–3.12)	1.46	(0.76–2.82)
*P*_trend_					0.21		0.39		57	0.77		0.98			0.12		0.22	
Continuous (25 g/day intake increment)					1.06	(0.97–1.15)	0.95	(0.82–1.09)		0.95	(0.81–1.12)	0.94	(0.79–1.11)		1.00	0.76–1.32)	0.95	(0.72–1.27)

^a^Adjusted for age (yrs) and sex.

^b^Adjusted for age (yrs), sex, total energy intake (kcal), cigarette smoking (never, ever, current), alcohol consumption (g/day), BMI (kg/m2), non-occupational physical activity (<30, 30–60, 60–90, >90 min/d), and level of education (lower vocational, second and medium vocational, university and higher vocational). If applicable, additionally adjusted for complementary meat groups holding total meat constant.

^c^Intake based on raw meat weight.

^d^Categories of intake 0, >0−<6.6, ≥6.6−<22.5, and ≥22.8 g/day.

^e^Categories of intake: 0, >0−<10, ≥10−<20, and ≥20 g/day.
